# Preservation of Complex Multiphase Architectures in Polymer‐Based Artificial Cells by Photo‐Crosslinking

**DOI:** 10.1002/advs.202514449

**Published:** 2025-11-21

**Authors:** Madelief A. M. Verwiel, Alexander B. Cook, Yiǧitcan Sümbelli, Nadia A. Erkamp, Jan C. M. van Hest

**Affiliations:** ^1^ Bio‐Organic Chemistry, Institute for Complex Molecular Systems (ICMS) Eindhoven University of Technology Eindhoven 5600MB The Netherlands

**Keywords:** artificial cells, coacervates, photo‐crosslinking, multiphase, internal structure, (dynamically induced) phase separation

## Abstract

Nature utilizes phase separation to create biomolecular condensates which spatially organize cellular components. In artificial cells, however, mimicking the structure of cellular multiphase systems is challenging. While transient or dynamically induced structures can be created, their lifetime is often very limited. Here, a strategy is presented for stabilizing these structures via photo‐crosslinking. Specifically, methacrylate groups are incorporated in one of the scaffold polymers of this coacervate‐based artificial cell. UV light is applied directly after the formation of the transient structure, allowing for dynamic formation but at the same time extending the structure's lifetime by at least 5–10 times. Interestingly, this strategy is effective on single and multiphase condensates. Additionally, spatio‐temporal control over irradiation can create samples in which condensates and parts of condensates have different structures, achieving programmable asymmetry which is rarely observed in artificial systems. It is expected that creating long‐lived intricately structured condensates will contribute to performing increasingly complex functions and develop more advanced artificial cells.

## Graphical abstract

Phase separation organizes cellular components, yet stabilizing such dynamic structures in artificial cells remains challenging. A photo‐crosslinking approach is presented that prolongs the lifetime of different structures in coacervate‐based artificial cells while maintaining dynamic formation. Spatially controlled irradiation yields asymmetric, long‐lived multiphase structures, with enhanced robustness, advancing design strategies for functional and programmable artificial cells.

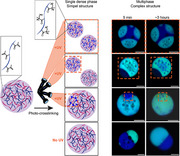

## 1 Introduction

Nature employs phase separation and biomolecular condensates as tools for spatial organization of biomacromolecular components and organelles.^[^ ^1^, ^2^ ^]^ Often, this process leads to the formation of interconnected multiple phases with interesting morphologies. For example, the nucleolus of eukaryotic cells contains core‐shell or nested microstructures where one phase is contained within another phase.^[^ ^3^ ^]^ The bulk of the nucleolus is the granular component (GC), while ribosomal genes and RNA are transcribed in small phases of the nucleolus called the fibrillar centre (FC) and dense fibrillar component (DFC).^[^ ^3^ ^]^ Multiphase structures in which boundaries are in contact with each other are also known, such as the interaction between P‐bodies and stress granules in the cytoplasm of cells which can include docking and fusion.^[^ ^4^ ^]^ Multiphase morphologies thus have structures which are intertwined with their function; partitioning biomolecules and regulating the concentration or activities of components.^[^ ^5^ ^]^ Mimicking the structure of cellular multiphase systems is challenging especially when preserving complex and dynamic structures over longer timescales.^[^ ^6^, ^7^, ^8^, ^9^, ^10^, ^11^ ^]^

Phase separation is driven by the balance of enthalpy and entropy of the interacting molecular components.^[^ ^1^ ^]^ The transition from dilute to dense phase is a dynamic equilibrium toward energy minimization affected by macroscopic factors such as temperature, pH, and salt concentration. The inhibition of this dynamicity can lead to disregulation and disease or it could be a tool to potentially treat disease in other cases.^[^ ^12^, ^13^, ^14^ ^]^ Developing new techniques to maintain dynamic or transient condensates would be beneficial for studying model systems of disease, and for advancing artificial cell systems.

In the artificial cell field, researchers have been investigating multiphase structures with a variety of materials.^[^ ^15^ ^]^ Artificial cell research has emerged as a way to unravel and reproduce hallmarks of active living systems, which involve aspects such as compartmentalization, growth and division, information processing, energy transduction, and adaptability (i.e., mobility). Although the ultimate goal of this field may be to create compartments that accommodate all aspects of living cells, thereby reproducing life, the development of artificial cell models provides additional value through the study of biological processes in a controlled environment.^[^ ^2^ ^]^ Our group has developed artificial cells based on a semi‐synthetic polymer coacervate system,^[^ ^16^, ^17^, ^18^ ^]^ which has been shown to form internal multiphase structures upon addition of additional charge species.^[^ ^19^ ^]^ Others have developed frameworks for understanding such multiphase separation in synthetic charged polymer systems,^[^ ^20^, ^21^ ^]^ which has been a great advancement in our understanding of the fundamental interactions in biomolecular condensates and other phase separating systems. Likewise, polypeptides and proteins have also been investigated as models for studying multiphase separation, where it has been shown that polypeptide chain length and sequence can play an important role in dense phase characteristics.^[^ ^22^, ^23^, ^24^, ^25^, ^26^ ^]^ Nucleic acid multiphase coacervates can be produced with various RNA and DNA structures,^[^ ^27^, ^28^ ^]^ and can show phase specific accumulation of cargo molecules.^[^ ^29^ ^]^ However, these coacervate systems are typically thermodynamically unstable and are leading to dissolution or coalescence of phases, limiting their further studying or use for in depth analysis and application of complex multiphase structures in artificial cells.

Recently, it has been shown that incorporating photo‐crosslinkable groups into phase separating polymers allows for hydrogel formation which was used to study the biophysical cues of the extracellular matrix.^[^ ^30^ ^]^ Here, we incorporate methacrylate groups onto amylose‐based charged polymers. Upon generation of additional internal DNA phases, we can freeze the multiphase structures inside the complex coacervate artificial cell droplets with UV irradiation. The fluidity of the micrometer‐sized multiphase constructs is characterized with fluorescence recovery after photobleaching (FRAP) experiments. Our approach enables coacervate phase architecture to be maintained for at least 3 hours as compared to ∼0.5 hours for non‐photo‐irradiated samples. This strategy can also be applied to sub‐coacervate regions by utilizing a 405 nm laser of a confocal microscope to irradiate the desired region. Photo‐crosslinking also increases the robustness of the artificial cells and protects DNA being degraded by endonucleases. This study allows the construction of artificial cells with spatiotemporal control over subcellular multiphase separation patterns, which increases our capacity to design artificial cells with functional architectures.

## 2 Results and Discussion

### 2.1 Slowing Down Diffusion by Coacervate Crosslinking

**Figure 1:**
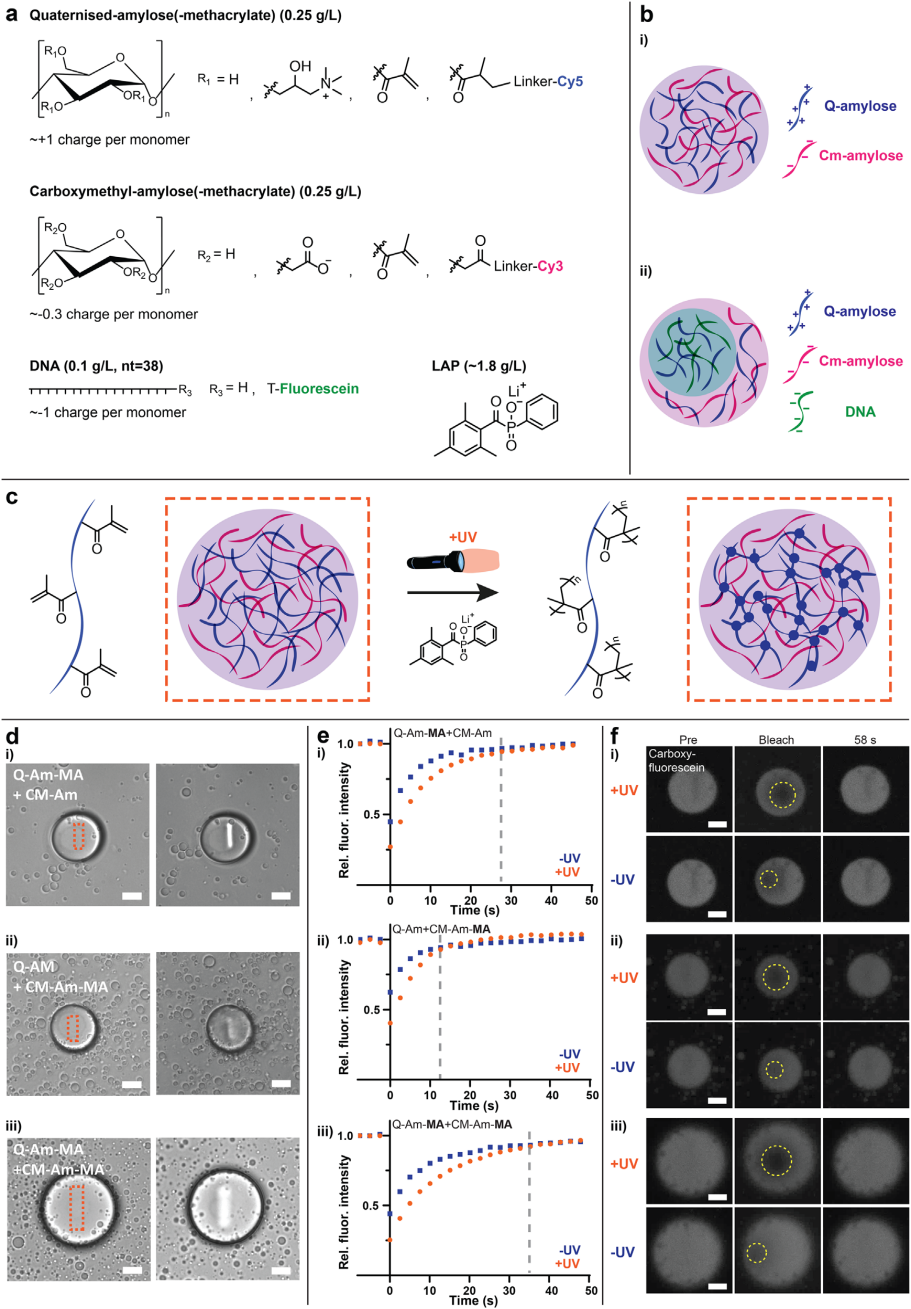
Polymer crosslinking in coacervates by introduction of methacrylate groups. a) Molecular structures of the main coacervate components Q‐amylose(‐MA), Cm‐amylose(‐MA), optional DNA and LAP photo initiator. b) Schematic of the single phase separated coacervates formed when Q‐Am(‐MA) and Cm‐Am(‐MA) are mixed (i), and the multiphase coacervate formed when mixing Q‐Am(‐MA), Cm‐Am(‐MA), and DNA (ii). c) When methacrylate groups are introduced to either one or both of the amyloses, the polymers can be crosslinked by the addition of a photo initiator and UV light (orange dashed square), leading to crosslinked coacervates. d) Local irradiation of coacervates with 405 nm laser light (100% laser power, 500 frames) changes the optical properties of the coacervates in bright field confocal microscopy. Methacrylate groups are introduced to either (i and ii) or both (iii) Q‐Am and Cm‐Am. e,f) Fluorescence Recovery After Photobleaching (FRAP) of fluorescein shows slower recovery for coacervates irradiated with 405 nm light. Methacrylate groups are introduced to either (i and ii) or both (iii) Q‐Am and Cm‐Am. Scale bars are 10 micrometer.

To create artificial cells with custom architectures, we use a complex coacervate system previously used in our group,^[^ ^16^, ^19^, ^31^, ^32^, ^33^ ^]^ to which we incorporate photo‐crosslinkable methacrylate (MA) groups. Our system contains two amylose (Am) compounds which have been quaternized or carboxymethylated to have a positive or negative charge respectively (**Figure** **1**a, Experimental Section). Additionally, a stabilizing terpolymer, salt and buffer are present (Experimental Section). The favorable electrostatic interaction between the positively charged Quaternized‐amylose (Q‐Am) and negatively charged Carboxymethyl‐amylose (Cm‐Am) promotes liquid–liquid phase separation, resulting in a condensed coacervate phase in a dilute phase (Figure 1b‐i). Additionally, negatively charged single stranded DNA is added to the system, which causes multiphase separation similar to previously described systems^[^ ^20^, ^34^ ^]^ (Figure 1b‐ii). The DNA has a higher density of negative charges than Cm‐amylose, causing the formation of two thermodynamically stable dense phases within the coacervate; a DNA‐poor and DNA‐rich phase.

Once we kinetically create more complex transient architectures by quickly changing salt or temperature, these newly formed architectures will be thermodynamically unfavorable because of the increase in surface area, and thus energy. This leads to short life‐times of the transient architectures which revert back to the thermodynamically stable state.^[^ ^19^ ^]^ To preserve the transient coacervate architectures, the rationale is to crosslink the amylose polymers, which limits molecular diffusion and creates a more robust artificial cell (Figure 1c).

To crosslink the amylose (Am) polymers, we incorporate methacrylate (MA) groups onto one or both of them (Quaternized‐amylose‐methacrylate (Q‐Am‐MA) and Carboxymethyl‐amylose‐methacrylate (Cm‐Am‐MA), Figure S1, Supporting Information, Experimental Section), which can undergo a radical addition reaction when initiated by external radicals. These radicals are provided by UV mediated cleavage of the photoinitiator LAP (lithium phenyl‐2,4,6‐trimethylbenzoylphosphinate). The occurrence of a crosslinking reaction after localized irradiation with a 405 nm laser was confirmed by a visible change in light refraction, as observed by bright field confocal microscopy (Figure 1d, orange dashed boxes indicate 405 irradiated region). To establish if the diffusion rates within the crosslinked coacervates were changed, FRAP was executed on carboxyfluorescein in coacervates containing the methacrylate groups on the Q‐amylose (Q‐Am‐MA & Cm‐Am)(i), Cm‐amylose (Q‐Am & Cm‐Am‐MA)(ii), or both Q‐ and Cm‐amylose (Q‐Am‐MA & Cm‐Am‐MA)(iii), before and after irradiation with UV light (Figure 1e,f). Coacervates containing the methacrylated amyloses (x‐Am‐MA) show slower fluorescence recovery after UV irradiation, indicating that the amylose polymers are (partly) crosslinked. The biggest difference in recovery can be observed when both charged amyloses are crosslinked (Q‐Am‐MA & Cm‐Am‐MA). The fluorescence signal recovers faster in coacervates with Cm‐Am‐MA than with Q‐Am‐MA, which can be explained by the difference in degree of substitution of the amyloses with methacrylate groups, which is 0.18 and 0.27 for Cm‐Am‐MA and Q‐Am‐MA respectively. The higher the degree of substitution for the methacrylates, the more crosslinking can be obtained, and the slower the fluorescence recovery in FRAP. Altering the degree of substitution of the methacrylate groups thus provides a way to tune the amount of fixation of the coacervates. So, to create artificial cells with custom architectures we will use the photo‐crosslinkable methacrylated amyloses in our synthetic cells. From here on, coacervates are used which have a methacrylated Q‐amylose (Q‐Am‐MA) and a non‐methacrylated Cm‐amylose (Cm‐Am), since this yielded intermediate FRAP recovery times and thus might provide Q‐Am‐MA fixation without losing full coacervate dynamicity.

### 2.2 Formation and Preservation of Complex Architectures

**Figure 2:**
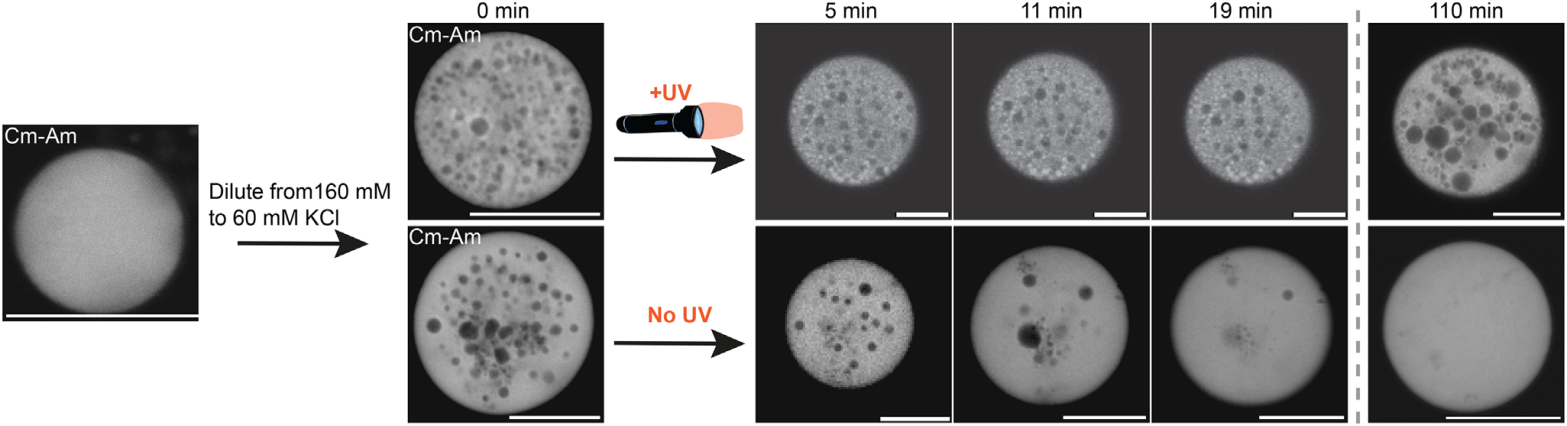
Preservation of complex architectures in coacervates. Rapid dilution of a coacervate sample from 160 to 60 mM KCl nucleates dilute phase droplets in the dense phase coacervates. This is a transient architecture in which the dilute phase droplets coalesce and fuse with the bulk dilute phase. However, when the coacervates contain methacrylate groups and are irradiated with 365 nm light for 10 minutes (in the presence of a photo initiator), the Q‐Am‐MA is crosslinked, leading to the preservation of the complex architecture for ar least 110 minutes, whereas the complex architecture is lost after ∼20 minutes when no UV light is shone. Scale bars are 20 µm. Note, different coacervates from the same samples are shown at 110 min than for 5, 11, and 19 min. Quantification of de dilute phase fractions at 5, 11, and 19 min is provided in Figure S2b (Supporting Information).

To achieve preservation of transient structures in artificial cells, we form coacervates with complex architectures, which we aim to maintain by photo‐crosslinking of the Q‐amylose‐methacrylate polymer. Internal structures in coacervates can be formed by dynamically induced phase separation.^[^ ^9^, ^19^ ^]^ This is a kinetic process that results from a change in coacervate composition and results in nucleation of dilute and/or dense phase droplets, of which the formation is dependent on the rate of the composition change, the diffusion rates within the coacervates, and the coacervate size. By changing the interaction strengths of the phase separating polymers, e.g., by changing salt concentration, pH, or temperature, the phase diagram changes, leading to a different coacervate composition. To facilitate the change in coacervate composition when the conditions change abruptly, while having limited diffusion rates, droplets of the dilute phase can be nucleated in the dense coacervate phase. In our system, this can be accomplished by lowering the salt concentration from 160 to 60 mM KCl, which increases the interaction strength between the two charged amylose polymers (**Figure** **2**). However, this newly created architecture is a transient state that will dissipate over time to its lowest thermodynamical energy state, which resembles the homogeneous architecture before dilution of the salt. This happens through coalescence of the dilute phase droplets and their fusion with the dilute bulk phase (Figure 2, bottom row). To change the transient character of the complex architecture to a more permanent one, crosslinking of the amylose polymers offers a solution. After irradiation with UV light, coacervates with methacrylates on the Q‐Am preserve the complex architecture up to at least 110 minutes, whereas coacervates without UV irradiation lose the complex architecture after ∼20 minutes. Fusion of the dilute phase droplets can still be observed in the crosslinked coacervates, showing the dynamicity of the system, yet at a much slower rate than for the non‐crosslinked system. Only UV irradiation, without the change in salt concentration, does not yield different architectures (Figure S2a, Supporting Information). These results show that transient structures in our artificial cells can be preserved by photo‐crosslinking of the Q‐amylose‐methacrylate polymer.

### 2.3 Spatial Control Over Polymer Crosslinking in Multiphase Coacervates

**Figure 3:**
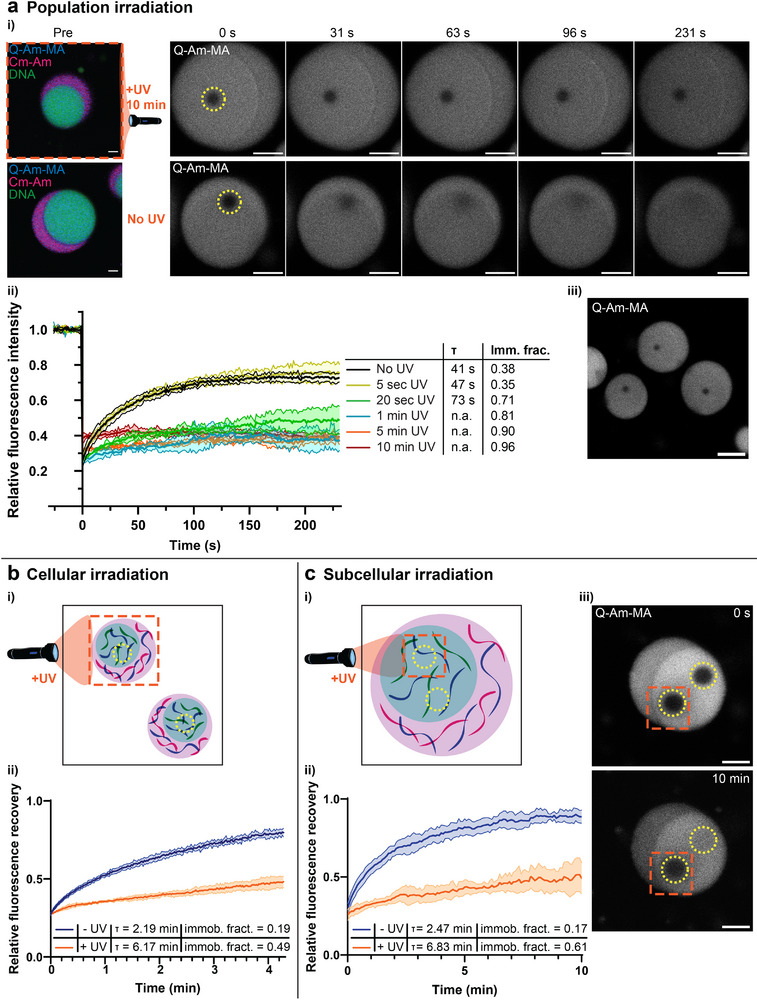
Spatial control over polymer crosslinking in multiphase coacervates. a) FRAP images (i) and data (ii) for multiphase coacervates containing Q‐Am‐MA with and without 365 nm light irradiation on a full coacervate population (overview images are provided in Figure S3). FRAP was performed on the Cy5 dye which is covalently linked to the Q‐Am‐MA. The fitting of the data yields the recovery half‐life τ and immobile fractions (Imm. frac.), both of which increase with increasing irradiation times. N=3 for all irradiation times. (iii) shows bleached regions in FRAP on three separate coacervates in one sample, after 10 minutes UV light irradiation on the full coacervate population. At the moment of imaging, it was 20 minutes after bleaching of the first coacervate, 12 minutes after bleaching of the second coacervate, and 5 minutes after bleaching of the third coacervate. No recovery of the fluorescent signal is observed on this time scale. b) i) Schematic showing local cellular irradiation with 405 nm light (orange dotted square) and subsequent FRAP (yellow dotted circles) in multiphase coacervates. ii) FRAP data on coacervates with and without UV irradiation, as indicated by the yellow circles in i). N=4. c) i) Schematic showing local subcellular irradiation with 405 nm light (orange dotted square) and subsequent FRAP (yellow dotted circles) in multiphase coacervates. ii) FRAP data on coacervates with and without UV irradiation, as indicated by the yellow circles in i). N=5. An example of FRAP images after 0 seconds and 10 minutes is provided in iii). Scale bars are 5 µm. Extra FRAP images after cellular and subcellular irradiation can be found in Figure S4 (Supporting Information).

Next, we aim for preservation of structures in multiphase coacervates, over which we also have spatial control on a population, cell and sub‐cellular scale. In addition to the single dense phase, multiphase coacervates can also be formed, by introducing 38nt ssDNA to the amylose‐based system (Figure 1b‐ii). Differences in the favorable electrostatic interaction between the positively charged Q‐amylose with negatively charged Cm‐amylose and DNA causes multiphase separation, resulting in the formation of two dense phases within the condensate, a DNA‐poor and DNA‐rich phase. The Q‐amylose and DNA have the highest concentration in the DNA‐rich phase, while the Cm‐amylose is most abundant in the DNA‐poor phase (**Figure** **3**a‐i, Pre).^[^ ^19^ ^]^ When introducing the methacrylate groups to the Q‐Am in this multiphase coacervate, again the Q‐Am‐MA is crosslinked by UV light. First, population‐wide irradiation was performed by using a 365 nm LED light for different amounts of time, after which the effect on the Q‐Am‐MA‐Cy5 diffusion in the coacervates was investigated by FRAP (Figure 3a). FRAP images (Figure 3a‐i) and data (Figure 3a‐ii) show that without UV irradiation the Q‐Am‐MA‐Cy5 signal almost fully recovers within 230 seconds, while after 10 minutes of UV irradiation no Q‐Am‐MA‐Cy5 signal is recovered in 230 seconds. Next to this, both the recovery half‐life τ and immobile fractions increase with increasing UV irradiation times, as can be expected. Already after 20 seconds of UV irradiation with the 365 nm LED, there is a significant increase in the fluorescence recovery half‐life and immobile fraction. After 10 minutes of irradiation, the immobile fraction reaches 0.96, indicating that the Q‐Am‐MA loses its dynamicity. FRAP recovery times differ from Figure 1 because of the bleaching of a different fluorescent molecule (attached to the amylose or loose) and use of a different light source for different amounts of time, showing that the amount of fixation can be regulated by different amounts of energy supplied to the system.

Next, in the context of artificial cells, one may also be interested in creating artificial tissue made up of cells with different properties. Single condensate crosslinking can be achieved by irradiating only the desired condensate at 405 nm (Figure 3b). The fluidity of the irradiated region was investigated by FRAP on Q‐Am‐MA‐Cy5 which for single cell irradiation, shows a 2.8 times longer recovery half‐life and 2.6 times larger immobile fraction after UV irradiation as compared to samples without UV light (Figure 3b‐ii; Figure S4, Supporting Information).

Lastly, one may be interested in crosslinking and stabilizing architectures in part of the cell/condensate (Figure 3c). For subcellular irradiation we find a 2.8 times longer recovery half‐life and a 3.6 times larger immobile fraction after UV irradiation. FRAP images after 0 seconds and 10 minutes also visually show that the irradiated region (orange box) of the coacervate shows very little fluorescent recovery after bleaching, while the non‐irradiated part fully recovers (Figure 3c‐iii; Figure S4, Supporting Information), indicating successful subcellular crosslinking. Thus, photo‐crosslinking in multiphase coacervates is successful with spatial control on a population, cell and sub‐cellular scale. We will use this control for preservation of complex architectures in multiphase coacervates in the next section.

### 2.4 Spatial Control Over Preservation of Complex Architectures in Multiphase Coacervates

**Figure 4:**
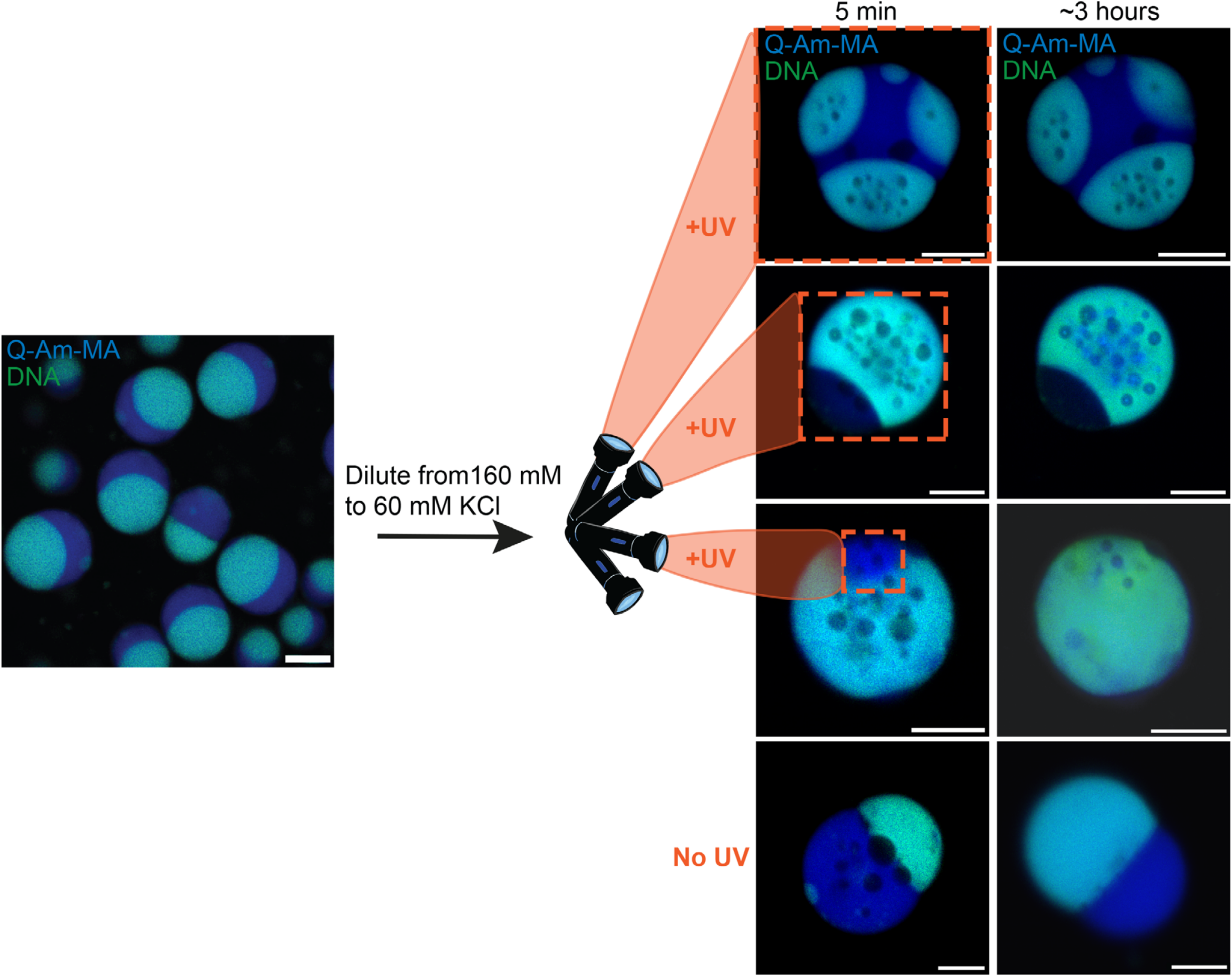
Spatial control over preservation of complex architectures in multiphase coacervates. Rapid dilution of a coacervate sample from 160 to 60 mM KCl nucleates dilute phase droplets in the dense phase coacervates. This is a transient architecture in which the dilute phase droplets coalesce and fuse with the bulk dilute phase. However, when the coacervates with complex architectures are irradiated with either 365 or 405 nm light (orange dashed box) (in the presence of a photo initiator), the Q‐Am‐MA can be crosslinked, leading to the preservation of the complex architecture for at least ∼3 hours. Spatial control over the irradiation, by using a 405 nm laser light in a confocal microscope, enables the fixation of complex subcellular structures. Scale bars are 10 µm.

Having spatial control over crosslinking of (one of) the amylose polymers also gives us control over the preservation of complex architectures. Also for multiphase coacervates, complex architectures can be created by a fast change in coacervate composition by changing a parameter that influences the polymer interaction strengths. By lowering the KCl concentration from 160 to 60 mM, dilute phase droplets are formed in the dense phase coacervate, mostly in the DNA‐rich phase. This is different from what has been reported before, where lowering the KCl concentration in amylose and DNA based multiphase coacervates did not only nucleate the dilute phase but also nucleated the DNA‐rich phase in the DNA‐poor phase and vice versa. This change in behavior can be explained by the addition of the methacrylate groups to the Q‐Am‐MA, which makes the polymer slightly more hydrophobic and changes the interaction strengths between the three charged polymers which alters the phase diagram. A similar effect, showing nucleation in fewer phases than described before, was also observed for changing the temperature (Figure S5, Supporting Information). The created complex architectures in the multiphase coacervates were irradiated with UV light at coacervate population, cellular, or subcellular level, freezing the complex architectures at these levels (**Figure** **4** top to bottom; Figure S6, Supporting Information). When no UV light is shone, the complex architecture dissipates, and after 3 hours the coacervates structurally resemble the coacervates before dilution. With the UV irradiation however, either being 365 nm LED light (5 min) for population irradiation or 405 nm laser light (5 frames, 400 Hz) for (sub)cellular irradiation, the complex architectures are still present after 3 hours. At the cellular level, complex architectures were even observed after 17 hours (Figure S6a, Supporting Information). For the subcellular irradiation, it can be seen that only the irradiated, and thus crosslinked, region maintains the complex architecture, while the rest of the coacervate is transient in nature and loses the complex structure. These results show that complex structures in multiphase coacervates were preserved with spatial control by photo‐crosslinking. This spatial control enables to create desired coacervate architectures at different length scales. We can form heterogeneous internal structures – not just between coacervates, but also within them. The combination of fast multiphase separation and post‐hoc stabilization enables access to a diverse set of coacervate architectures with a life span that is sufficient to further exploit their rich phase behavior.

### 2.5 Photo‐Crosslinking of Coacervates Increases Robustness and Protects Against DNA Degradation by Endonuclease

To emphasize the value of crosslinking multiphase coacervates, next to maintaining complex architectures, we show increased local robustness and added functionality by protection of DNA in the condensate phase from degradation by a nuclease. First, crosslinking coacervates makes them more resistant to higher KCl concentrations (Figure S8, Supporting Information). Non‐crosslinked coacervates start to show a different interfacial angle between the two dense phases at 300 mM KCl, and are completely dissolved in 400 mM KCl because of the screening of the polymer charges by the salt. Crosslinked coacervates, however, show no morphological difference at 300 mM KCl. Increasing the KCl concentration even further to 600 mM KCl only dissolves the outer phase, which mostly contains non‐crosslinked Cm‐amylose. This shows robustness of the crosslinked phase, while maintaining the dynamic behaviour of the non‐crosslinked region.

**Figure 5:**
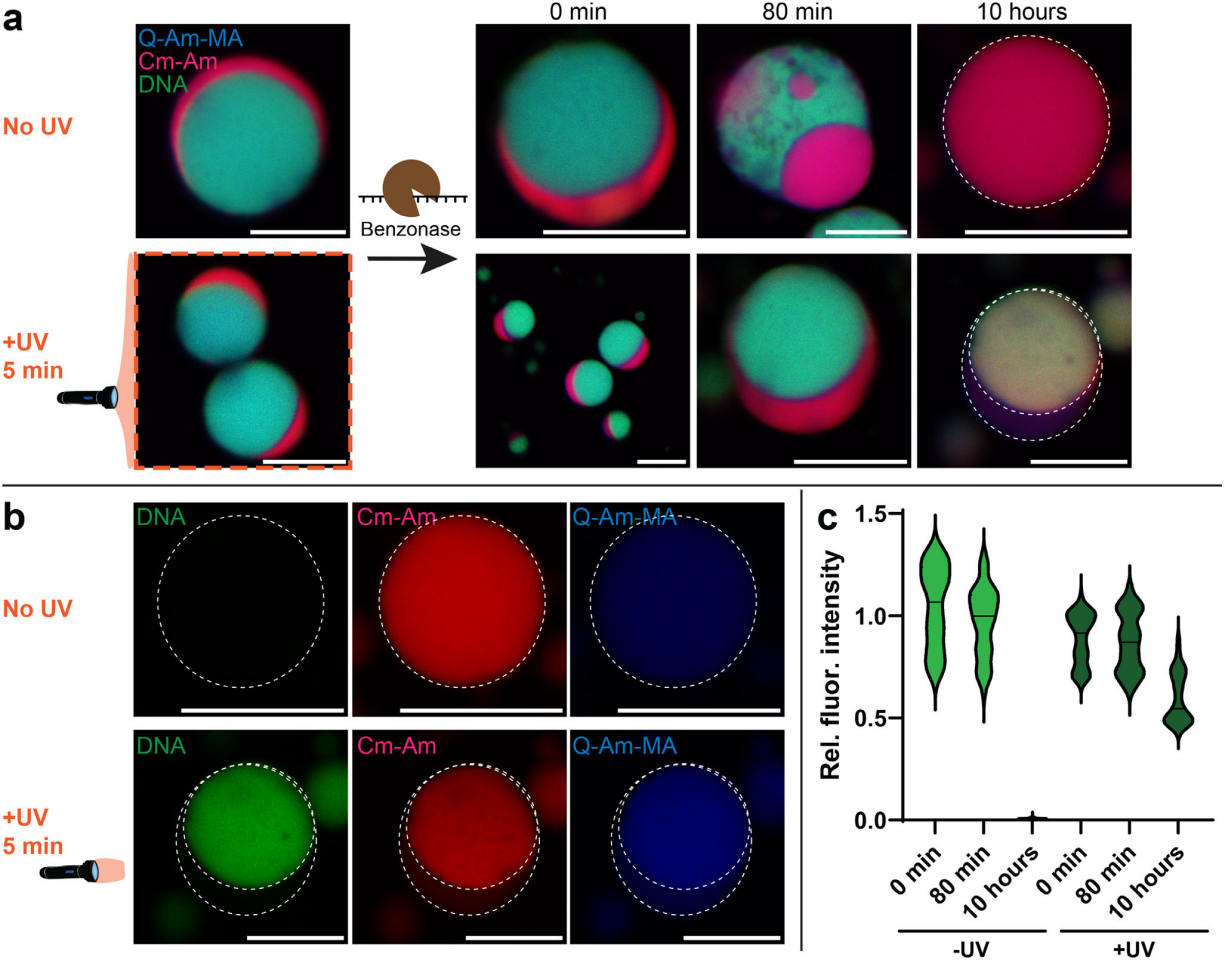
Photo‐crosslinking of coacervates protects against DNA degradation by nuclease. a) Multiphase coacervates containing Q‐amylose‐methacrylate, are either photo‐crosslinked or non‐crosslinked, after which endonuclease (Benzonase) is added. For the non‐crosslinked coacervates, DNA degradation is observed after 80 mins, and no DNA is visible after 10 hours. For the crosslinked coacervates, almost no difference is observed after 80 mins. After 10 hours, multiphasic coacervates are still observed. b) Separate fluorescence channels, 10 hours after Benzonase addition for crosslinked and non‐crosslinked coacervates. c) Quantification of the DNA intensity in the coacervates at 0 mins, 80 mins, and 10 hours after Benzonase addition. Scale bars are 10 µm. White dashed circles are a guide for the eye either showing one dense phase or multiphasic structures. Extra confocal images showing multiple coacervates, only the DNA signal, and controls after 24 hours can be found in Figure S7 (Supporting Information).

Next to this kind of robustness, we also show the protection of the DNA in the coacervates from degradation by Benzonase^®^ (Figure 5, Experimental Section). Crosslinking the Q‐amylose‐methacrylate slows down the degradation of the DNA in the coacervates by this nuclease. Without crosslinking, the first effects of the nuclease Benzonase degrading the DNA were visible after ∼80 minutes, showing a less homogeneous DNA‐rich dense phase. After 10 hours, the DNA intensity in the coacervates was almost completely absent and the remaining structure was a homogenous, simple dense phase, only containing Q‐amylose and Cm‐amylose (Figure 5b). For the crosslinked coacervates, the changes after 80 minutes of the Benzonase addition were minimal. After 10 hours there was a decrease in DNA intensity in the DNA‐rich phase, but it was still present (Figure 5b,c). Also, the structure of these coacervates was still multiphasic, and only became a single dense phase after 24 hours (Figure S7d, Supporting Information). It can be seen that the internal organization of the Cm‐amylose changes upon the degradation of (part of) the DNA. Where this Cm‐amylose was first predominantly located in the outer dense phase, it now was mostly present in the inner dense phase. Our hypothesis is that the degraded DNA fragments partly diffuse out of the coacervate, decreasing the internal DNA density and intensity. This decrease in negatively charged material in the inner phase, allows the negative Cm‐amylose to interact with the positively charged Q‐amylose which is mostly located in this inner phase. The decrease in DNA degradation by Benzonase after crosslinking can be explained by the limited diffusion of this enzyme into the coacervates. The crosslinking slows down the diffusion of larger molecules in and into the coacervates. This was also demonstrated by difference in uptake of three cargo molecules of different sizes, being Fluorescein, Fluorescein‐DNA, and GFP‐DNA in both crosslinked and non‐crosslinked coacervates (Figure S9, Supporting Information). The large GFP‐DNA, which is about the same molecular weight as Benzonase (∼27 kDa), showed no uptake in crosslinked coacervates (within 30 minutes), while it partitioned into non‐crosslinked coacervates. It can be hypothesized that Benzonase, in the same way, is not able to partition into the crosslinked coacervates on this timescale. Overall, this approach shows the effect of crosslinking on protecting DNA in the coacervates from an enzymatic degradation reaction, resulting in a prolonged multiphasic structure.

## 3 Conclusion 

Nature employs phase separation and biomolecular condensates as tools for spatial organization of cellular components and organelles. In artificial cells however, mimicking the structure of cellular multiphase systems is challenging especially when preserving complex and dynamic structures over longer timescales. This is because often used multiphase separated coacervate systems are thermodynamically unstable and progress toward dissolution or coalescence and fusion of phases. In this work we introduced photo‐crosslinkable methacrylate groups to our phase separating amylose polymers, which provides the ability to crosslink the amylose polymers by irradiation with UV light. By doing so, the fluidity of the coacervates decreases, as shown by FRAP, and complex architectures were maintained for at least 110 minutes, whereas in non‐irradiated coacervates the architecture dissipates to structurally simple coacervates after ∼20 minutes. The technique can also be applied to attain spatial control on a cellular and subcellular level by irradiation of the desired region. In this way, complex architectures in multiphase coacervates can be preserved for at least 3 hours at multiple length scales, while the complex architecture in non‐irradiated multiphase coacervates is lost at these timescales. This method stabilizes otherwise transient structures, pushing artificial systems closer to the features seen in biological ones. The photo‐crosslinking is selective and is spatially controllable, allowing for heterogeneous internal structures and programmable asymmetry which has not yet been shown in artificial cells. With this method, we are not just aiming to mimic structure, but we also see it as enhancing functionality and robustness, which are essential for synthetic biology. This was demonstrated by the salt tolerance and DNA protection as a result of photo‐crosslinking. Next to this, spatiotemporal control over subcellular multiphase separation patterns can be beneficial for studying model systems of disease, and for advancing artificial cell systems with functional architectures.

## 4 Experimental Section

### Materials

All chemicals were used as received unless otherwise stated. Poly(ethylene glycol) monomethyl ether 2kDa was purchased from RappPolymere, and trimethylene carbonate (1,3‐dioxan‐2‐one) was purchased from Actu‐All Chemicals. Amylose (12–16kDa) was supplied by Carbosynth and (3‐chloro‐2‐hydroxypropyl) trimethylammonium chloride (65 wt% in water) and methacrylic anhydride by TCI Europe. DBCO‐Cy3 and DBCO‐Cy5 were purchased from ThermoFisher. 38 nucleotide (nt) long ssDNA and HPLC purified Fluorescein‐labeled 38nt long ssDNA (5'TTTTTTTTTTCAGTCAGTCAGTCAGTCAGTCCATAAGG and complementary 5'CCTTATGGACTGACTGACTGACTGACTGAAAAAAAAAA) were ordered at Integrated DNA Technologies (IDT). DNA‐GFP was a kind gift by Indra van Zundert and was prepared as described in.^[^ ^19^ ^]^ All other chemicals and reagents were supplied by Sigma‐Aldrich.

### Synthesis and Modifications of Amyloses and Terpolymer

Amyloses were synthesized as described previously.^[^ ^16^ ^]^ Briefly, Q‐amylose was prepared via substitution with 3‐chloro‐2‐hydroxypropyltrimethylammonium chloride (Figure S1a‐i, Supporting Information). The degree of substitution was found to be 1.11 (Figure S1b‐i, Supporting Information). Cm‐amylose was prepared by addition of chloroacetic acid (Figure S1a‐ii, Supporting Information). The degree of substitution was found to be 0.47 (Figure S1b‐ii, Supporting Information). Terpolymer (triblock copolymer PEG‐PCLgTMC‐PGlu) was prepared as described previously,^[^ ^16^ ^]^ and dissolved in PEG (average molar mass 350).

Cm‐Am and Q‐Am were modified with methacrylate groups following a reported procedure.^[^ ^35^ ^]^ Q‐Am (75 mg, 0.243 mmol) was dissolved in 8 mL NaOH (pH 9.5), and an excess of methacrylic anhydride (375 mg, 2.43 mmol), compared to the amylose average repeat unit weight, was added (Figure S1a‐i, Supporting Information). The reaction was stirred at room temperature for 24 h, and was precipitated in cold ethanol. The resulting precipitate was washed with ethanol, re‐dissolved in Milli‐Q water, and dialyzed against water (3.5 kDa MWCO) before freeze drying. Cm‐Am was dissolved and a 10x excess of methacrylic anhydride, compared to the amylose average repeat unit weight, was added (Figure S1a‐ii, Supporting Information). The reaction was stirred at room temperature for 24 h, and was precipitated in cold ethanol. The resulting precipitate was washed with ethanol, re‐dissolved in Milli‐Q water, and dialyzed against water (3.5 kDa MWCO) before freeze drying. 1H Nuclear Magnetic Resonance (NMR) characterization data are presented in Figure S1b (Supporting Information). The degree of substitution was found to be 0.27 and 0.18 for Q‐Am‐MA and Cm‐Am‐MA, respectively.

### Formation of (Multiphase) Condensates

All amyloses were dissolved at a concentration of 1 mg/mL in buffer (20mM HEPES, 100mM KCl), pH 7.5. DNA was dissolved in Milli‐Q to reach a final concentration of 250 µM, of which 2.5 µM was labeled with fluorescein. To prepare condensates, buffer, Cm‐Am(‐MA), and Q‐Am(‐MA) were mixed whilst shaking at 1500 rpm at room temperature at an amylose charge ratio of 2.1:1 (Q‐Am(‐MA):Cm‐Am(‐MA)). For multiphase coacervates, ssDNA was premixed with the Cm‐AM, after which Q‐Am(‐MA) was added. After 2 minutes, LAP photoinitiator was added and after 6 minutes, terpolymer (50 mg/mL in PEG350) was added to the solution to stabilize the condensates. Samples were placed in an Ibidi slide with lid to prevent water evaporation.

### UV Settings Crosslinking

Crosslinking of assembled complex coacervate artificial cells was carried out on bulk coacervate samples in Ibidi microscopy slides (100 µL volumes in 18 well slides), with 365 nm UV light (CoolLED pE‐800 device, 50 mW mm^−2^) to create homogenously crosslinked coacervate droplets. For local irradiation, the Leica FRAP tool was used with a 405 nm laser (Diode laser, 50 mW mm^−2^). More information is provided in **Table** **1**.

**Table 1:** UV settings for crosslinking.

Figure	Light source	Wavelength	Irradiance	Irradiation time
Figure 1	Diode laser (Leica TSC SP8)	405 nm	50 mW mm^−2^ (100% laser power)	500 frames at 400Hz scan speed
Figure 2	CoolLED pE‐800	365 nm	50 mW mm^−2^	10 min
Figure 3a	CoolLED pE‐800	365 nm	50 mW mm^−2^	5 sec, 20 sec, 1 min, 5 min, 10 min
Figure 3b,c	Diode laser (Leica TSC SP8)	405 nm	50 mW mm^−2^ (50% laser power)	5 frames at 400Hz scan speed
Figure 4 (population)	CoolLED pE‐800	365 nm	50 mW mm^−2^	5 min
Figure 4 ((sub)cellular)	Diode laser (Leica TSC SP8)	405 nm	50 mW mm^−2^ (50% laser power)	5 frames at 400Hz scan speed
Figure 5	CoolLED pE‐800	365 nm	50 mW mm^−2^	10 min

### Confocal Microscopy

Images were taken with a Leica TSC SP8 confocal microscope equipped with a 405, 488, 552, and 638 nm laser, hybrid (HyD) and photomultiplier tube (PMT) detector, and HC PL APO CS2 20x/0.75 dry objective. Cooling and heating experiments were performed with a VaHeat system with precision single 5 x 5 mm smart subtrates (Interherence).

### Benzonase Endonuclease Addition

Benzonase endonuclease, suitable for biopharmaceutical production EMPROVE EXPERT, 100 000 U/vial, Order No.1.01695.0001 provided by Merck was used. 1µL of this Benzonase was added to 20µL coacervate samples in buffer (20 mM HEPES, 100 mM KCl) supplemented with 1.8 mM MgCl_2_. Benzonase Emprove Expert hydrolyzes internal phosphodiester bonds present between the nucleotides. Upon complete digestion, all free nucleic acids present in solution are reduced to 5'‐monophosphate terminated oligonucleotides, which are three to five bases in length. According to the supplier, the molecular weight of the Benzonase Emprove Expert is ∼27kDa.

### Analysis

Analysis was performed and figures were prepared using Fiji (2.3.051), GraphPad Prism 10, Adobe Illustrator (27.8.1).

### Immobile Fractions

Immobile fractions shown in Figure 3 were calculated by fitting the FRAP recovery curves, using the immobile fraction equation: Immobile fraction = (1‐plateau)/(1‐Y0), where Y0 is the Y value at X = 0.

### Statistics and Reproducibility

All confocal microscopy images show representative images. In Figure 1e,f, FRAP was done on three individual coacervates. Figure 3a‐ii, FRAP N = 3 for all irradiation times. Figure 3b‐ii FRAP N = 4 and Figure 3c‐ii FRAP N = 5 for both irradiated and non‐irradiated samples. In Figure 5c N⩾29 for every time point.

## Conflict of Interest

The authors declare no conflict of interest.

## Author Contributions

M.A.M.V., A.B.C., Y.S., J.C.M.H., and N.A.E. conceived the study. M.A.M.V., A.B.C., Y.S., and N.A.E. performed the investigation. A.B.C., J.C.M.H., and N.A.E. acquired funding. M.A.M.V., A.B.C., J.C.M.H., and N.A.E. wrote the original draft, and all authors reviewed and edited the paper.

## Supporting information

Supporting Information

## Data Availability

The data that support the findings of this study are available from the corresponding author upon reasonable request.
