# Supplementary material for: Preservation of Complex Multiphase Architectures in Polymer‐Based Artificial Cells by Photo‐Crosslinking
Source: Adv Sci (Weinh). 2025 Nov 21;13(16):e14449. doi: 10.1002/advs.202514449 (PMC13042627; doi:10.1002/advs.202514449)
Supplement: Supplementary file 1 — Supporting Information [file ADVS-13-e14449-s001.pdf]

Supplementary Figures

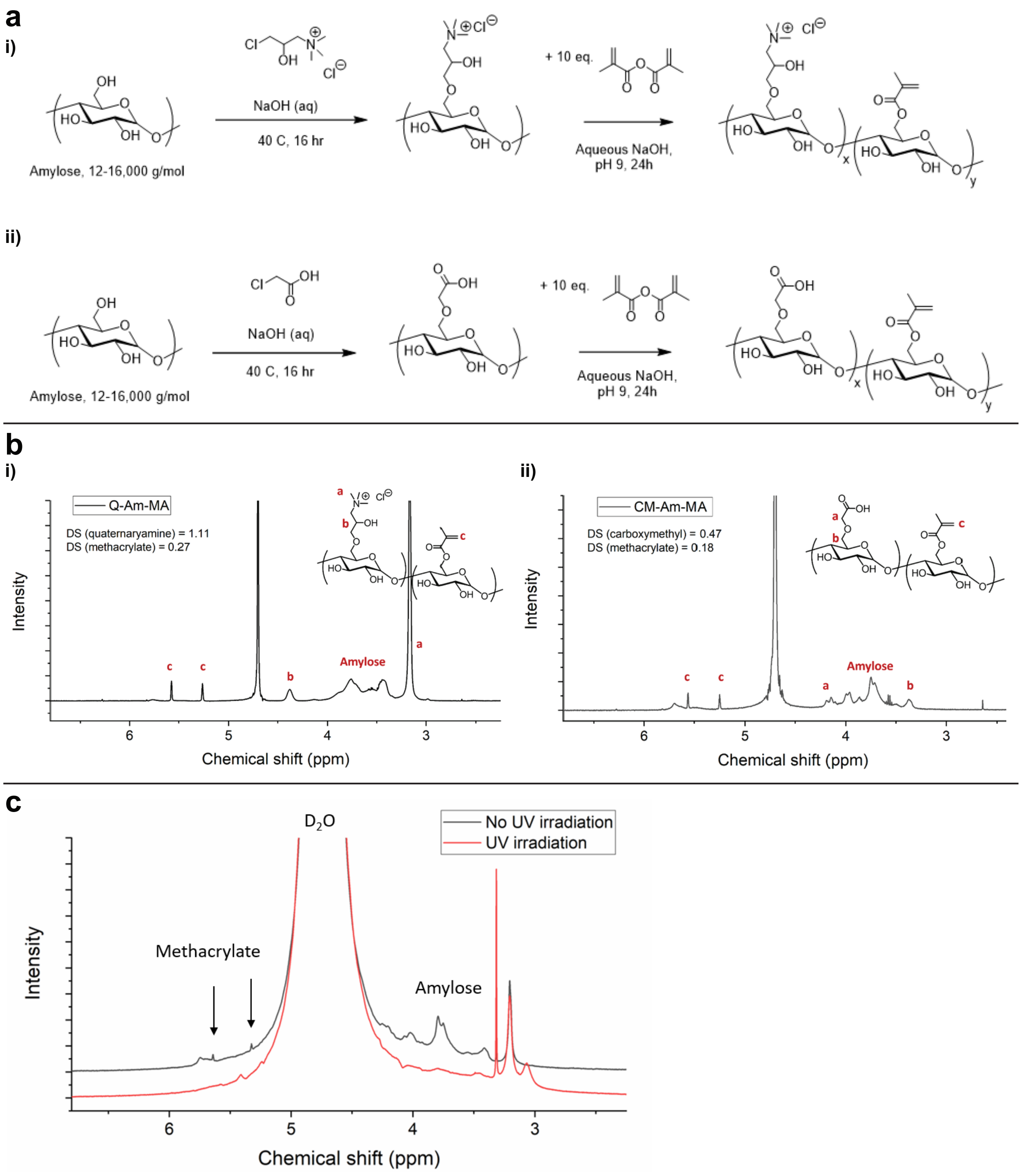

Figure S1. Synthesis and characterization of methacrylated amyloses. a Synthesis routes of i) Q-Am-MA and ii) Cm-Am-MA. **b** ^{1}H NMR characterization of i) Q-Am-MA, and ii) Cm-Am-MA. Degree of substitution (DS) of Q-Am-MA: quaternized amine = 1.11 and methacrylate = 0.27. DS of Cm-AM-MA: carboxymethyl = 0.47 and metacrylate = 0.18. **c** Mixture of Q-Am-MA and CM-Am at 10 mg/mL in D_2O:PBS = 50:50 before and after 10 min UV irradiation. No UV irradiation shows methacrylate peaks which disappear after UV irradiation.

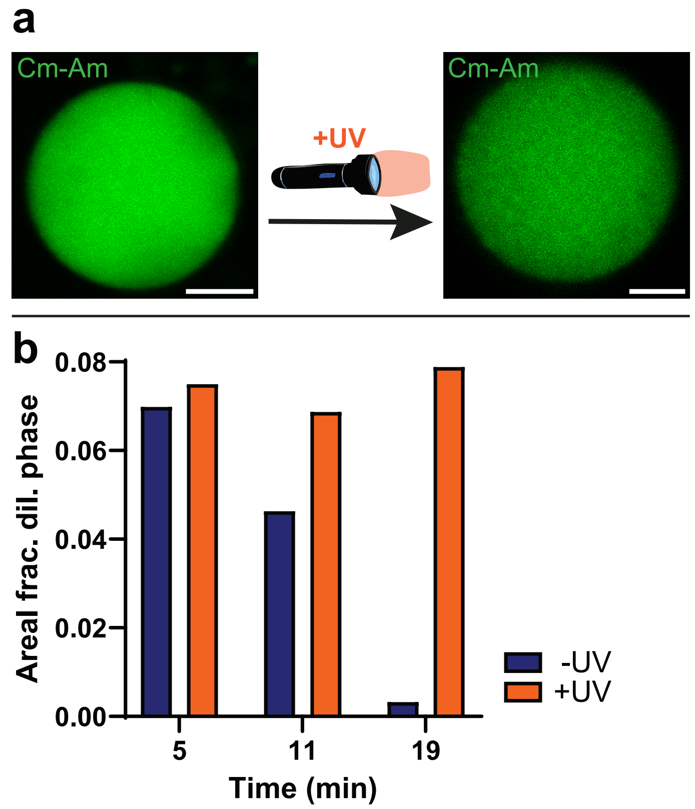

Figure S2. **a** UV irradiation yields no complex architectures. Note!: the green signal represents Cm-amylose as is indicated in the figure. Scale bars are 5 μm. **b** Quantification of the dilute phase fraction in the images of Figure 2 for 5, 11, and 19 minutes. When crosslinked, the dilute phase fraction does not change over these 19 minutes, while the dilute phase fraction approaches zero when the coacervates are not crosslinked.

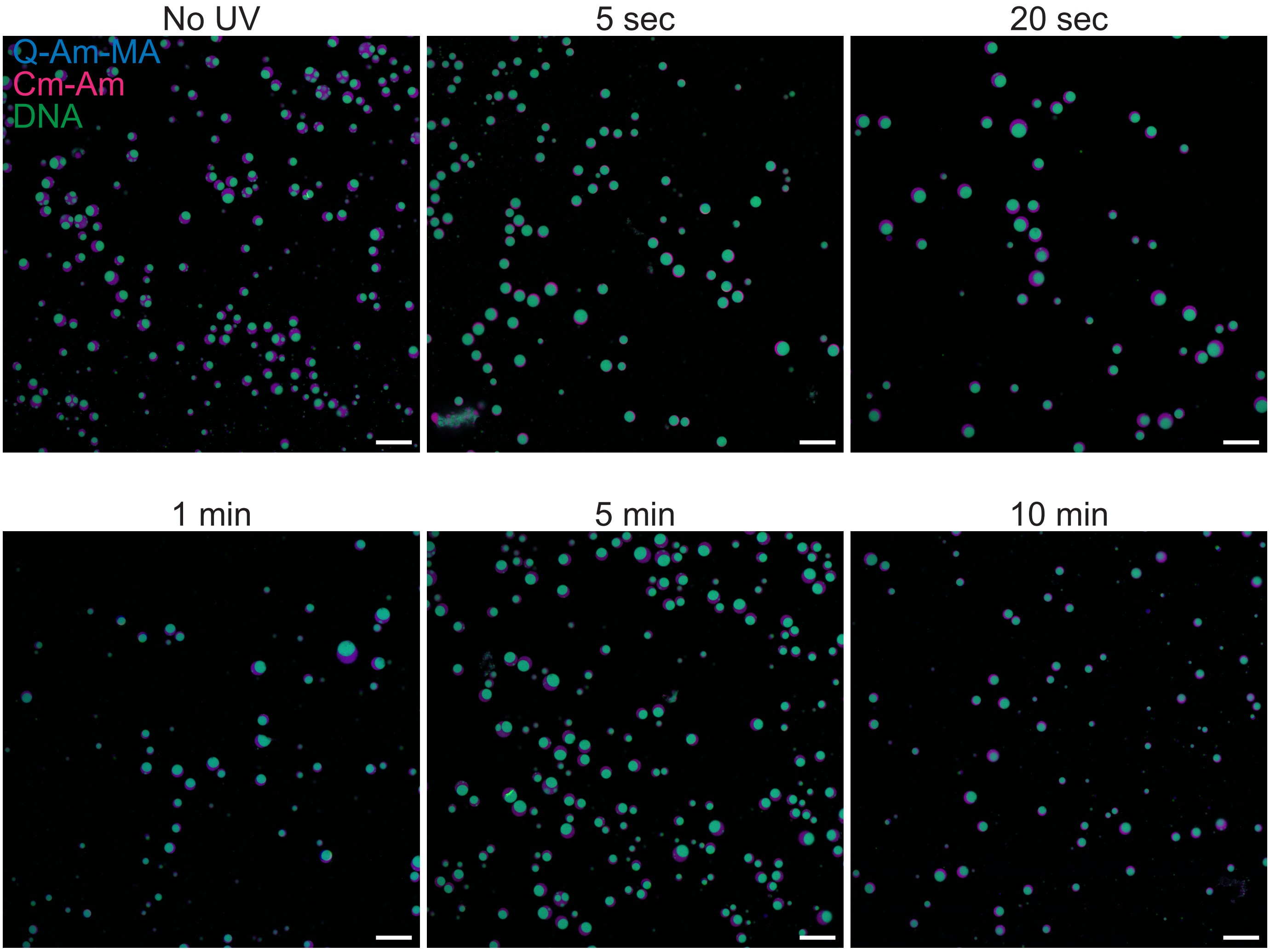

Figure S3. Confocal images population irradiation. Confocal images after the irradiation of the entire sample/population with UV light for different amounts of time. Scale bars are 50 μm.

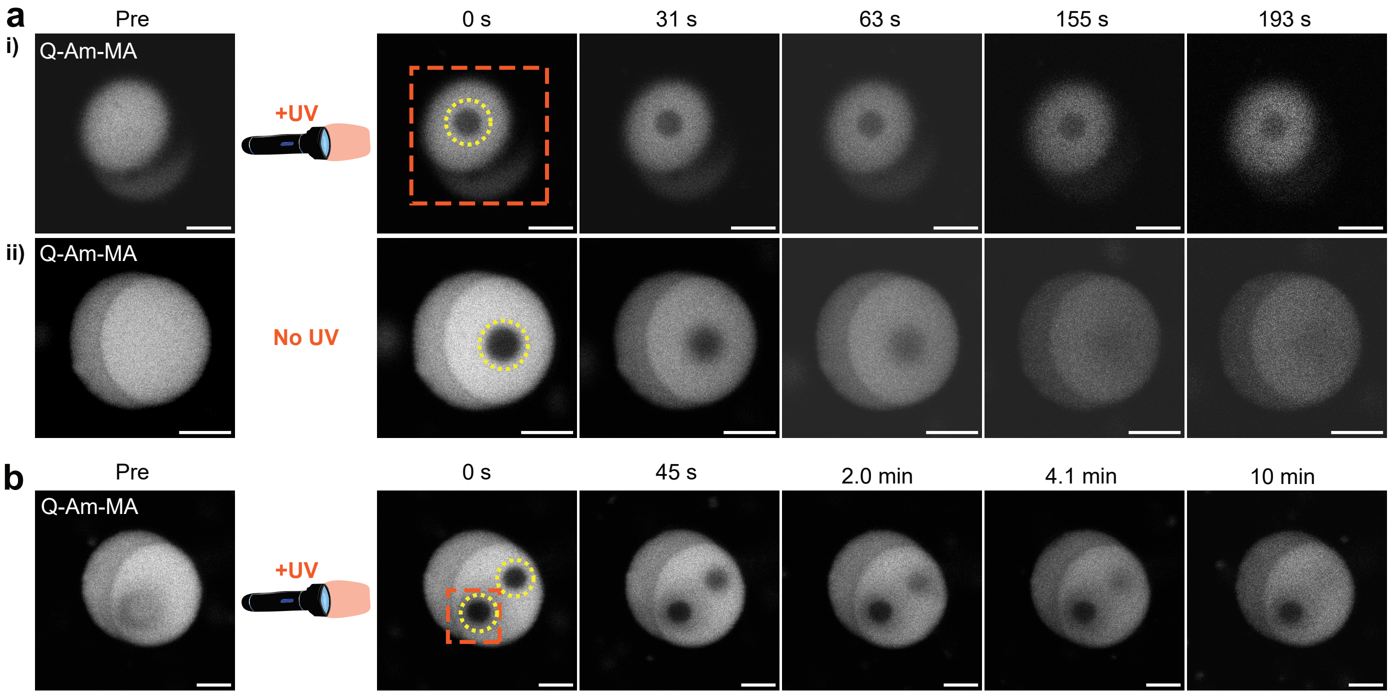

Figure S4. FRAP images (sub)cellular irradiation. **a** Cellular crosslinking. FRAP images after i) cellular irradiation with 405 nm laser light (orange dashed box), ii) no UV irradiation. **b** Subcellular crosslinking. FRAP images after subcellular irradiation with 405 nm laser light (orange dashed box). Scale bars are 5 μm, yellow dashed circles indicate bleached region in FRAP.

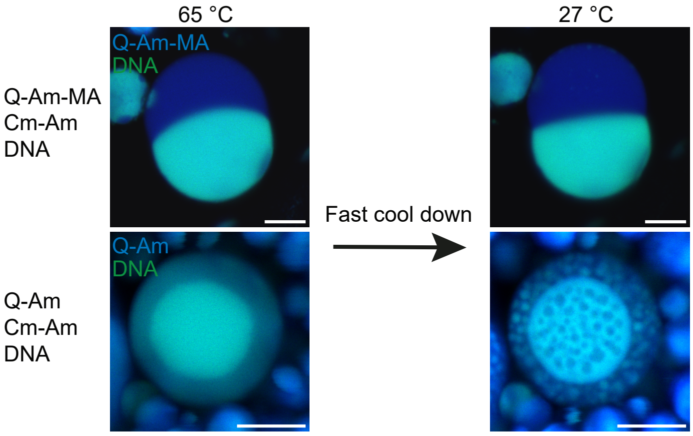

Figure S5. Structural change upon cooling coacervates from 65°C to 27°C. Cooling down coacervates with Q-Am-MA does not generate complex architectures. Cooling down coacervates with Q-Am nucleates the DNA-poor phase in the DNA-dense phase and vice versa, which is in line with perviously reported data [19]. Scale bars are 10 μm.

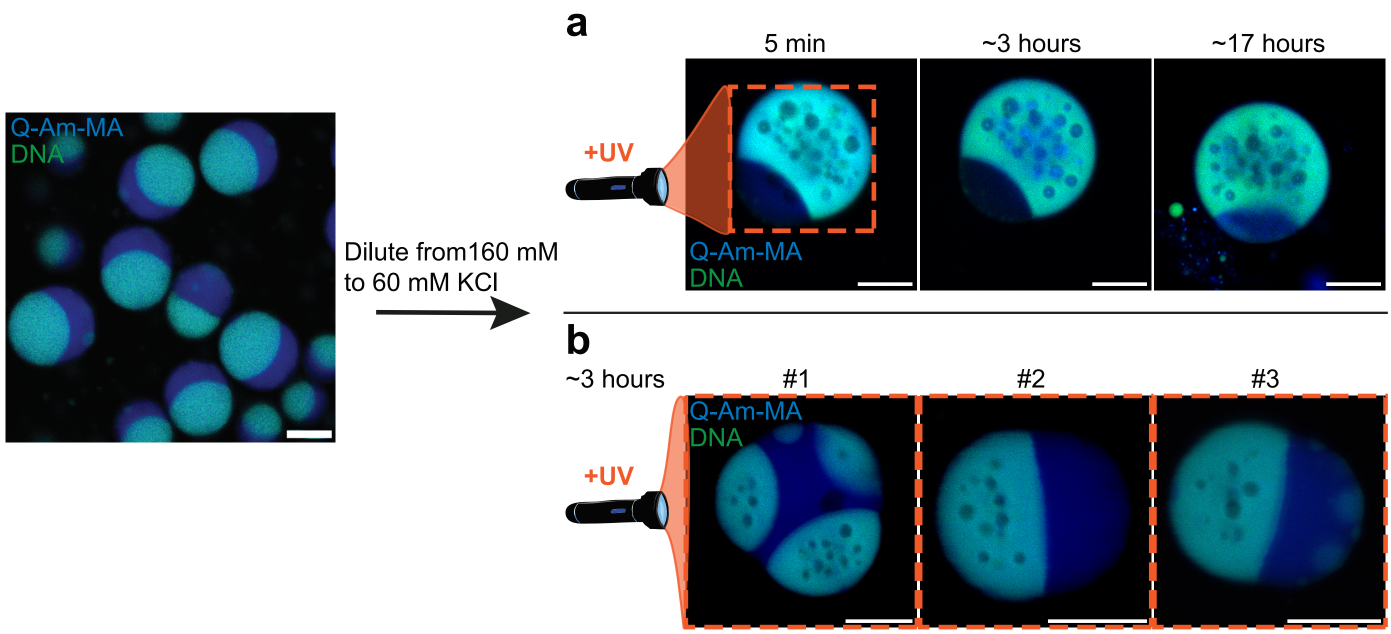

Figure S6. Preservation of complex architectures in multiphase coacervates: extra data. a After UV irradiation at the cellular level, complex architectures are preserved for at least ∼17 hours in multiphase coacervates. **b** Three examples of preservation of complex architectures in multiphase coacervates after ∼3 hours when irradiated at the coacervate population level. Scale bars are 10 μm.

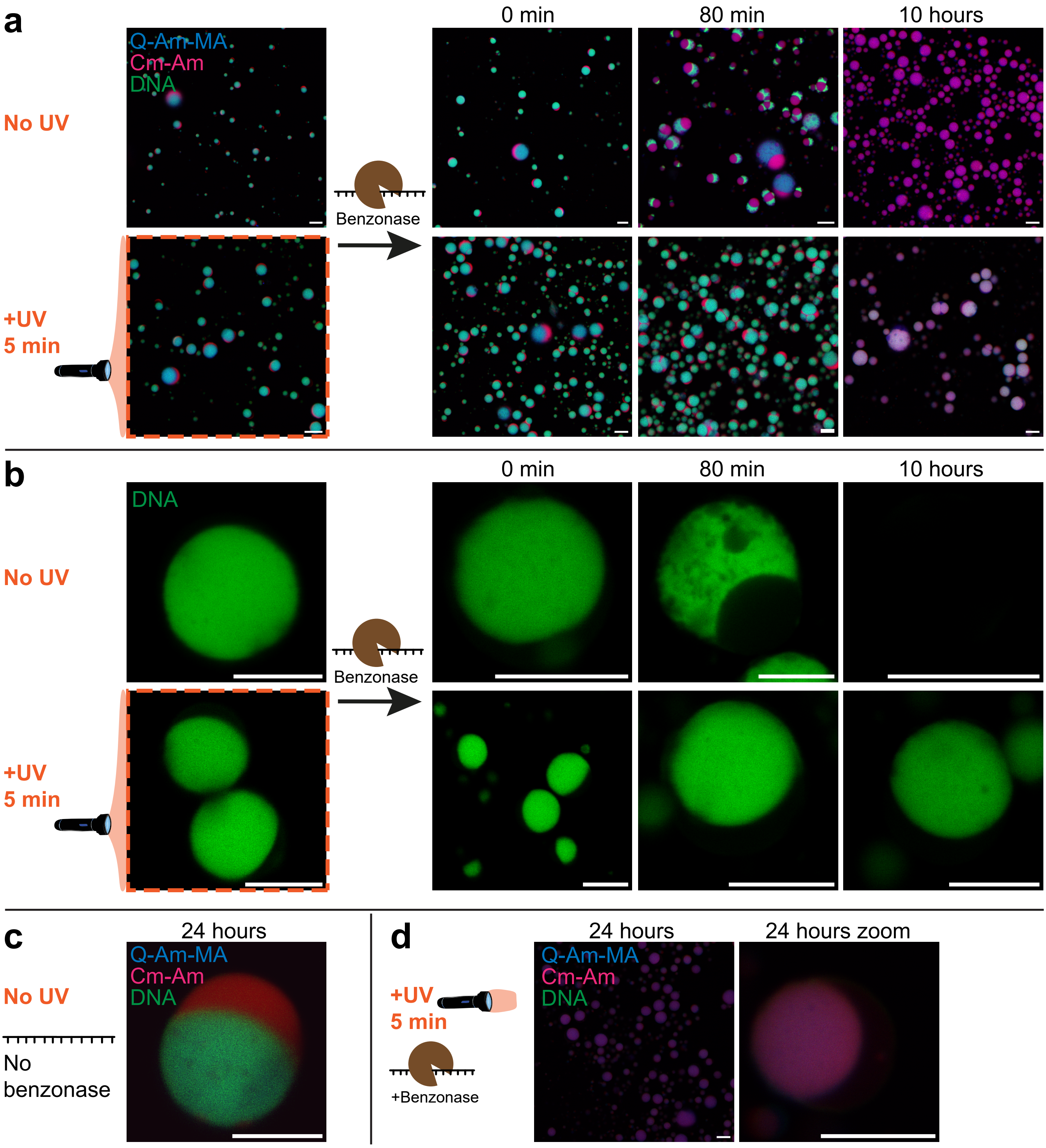

Figure S7. Photo-crosslinking of coacervates protects against DNA degradation by nuclease. a Uncropped confocal images of Figure 5a. **b** Confocal images as in Figure 5a only showing the DNA signal. **c** When not adding Benzonase and without crosslinking, the multiphasic structure is still present after 24 hours. **d** 24 hours after adding Benzonase to crosslinked coacervates, the structure of the coacervates is more single phase than multiphase. This resembles the structure at 10 hours for non-crosslinked coacervates. Scale bars are 10 μm.

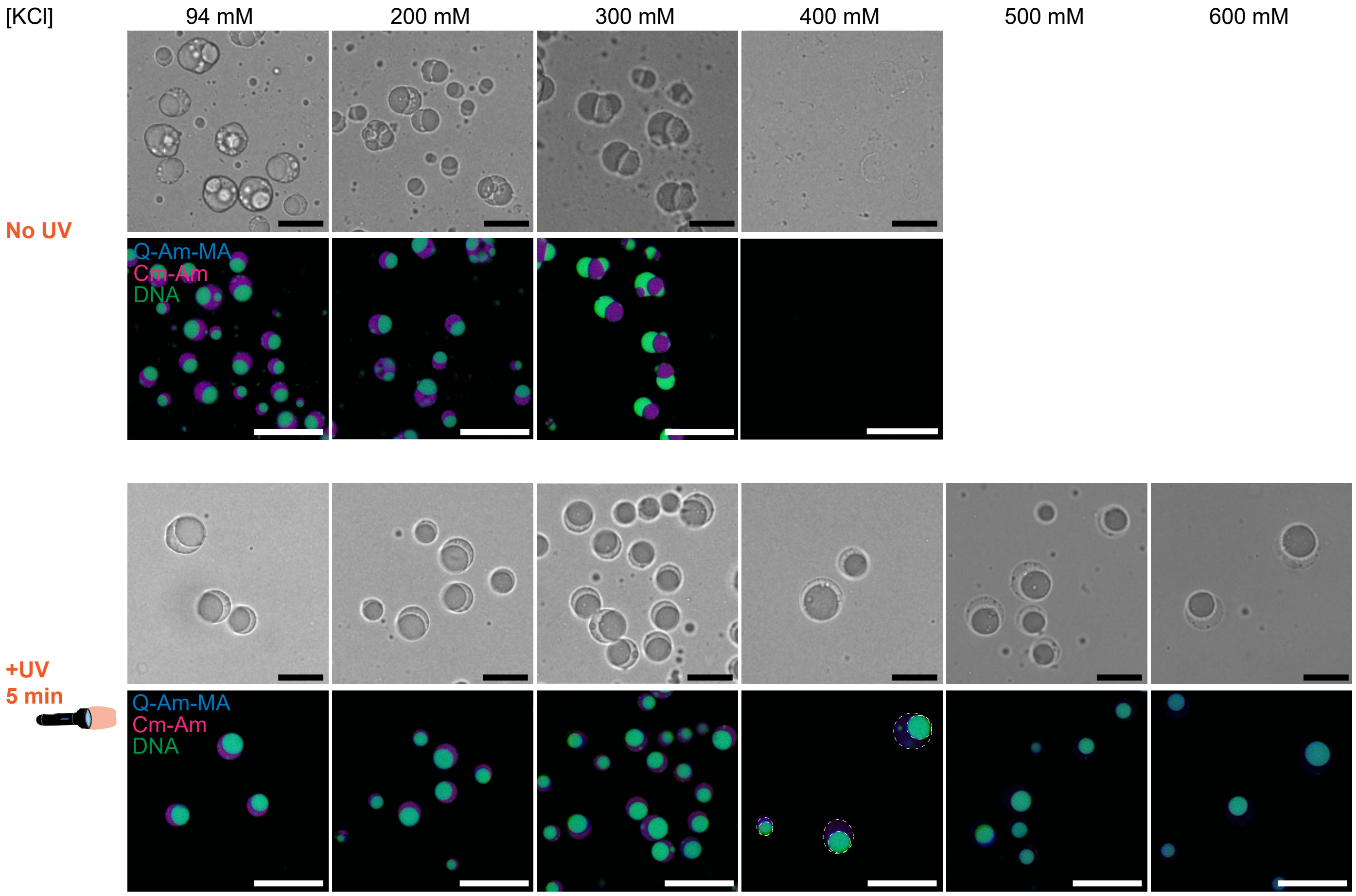

Figure S8. Crosslinking prevents coacervate dissolution at higher KCl concentrations. Without irradiating the coacervates with UV light, the contact angle of the two distinct phases starts to change at 300 mM KCl, and the coacervates dissolve completely at 400 mM KCl because of the screening of the charges on the polymers by the salt. After UV irradiation, coacervates remain present till at least 600 mM KCl. From 400 mM KCl on, the outer dense phase starts to dissolve. This is the less crosslinked phase, which mainly consists of Cm-Amylose. Crosslinking makes the coacervates more resistant to salt, but still shows dynamicity for the non-crosslinked materials. Scale bars are 20 μm.

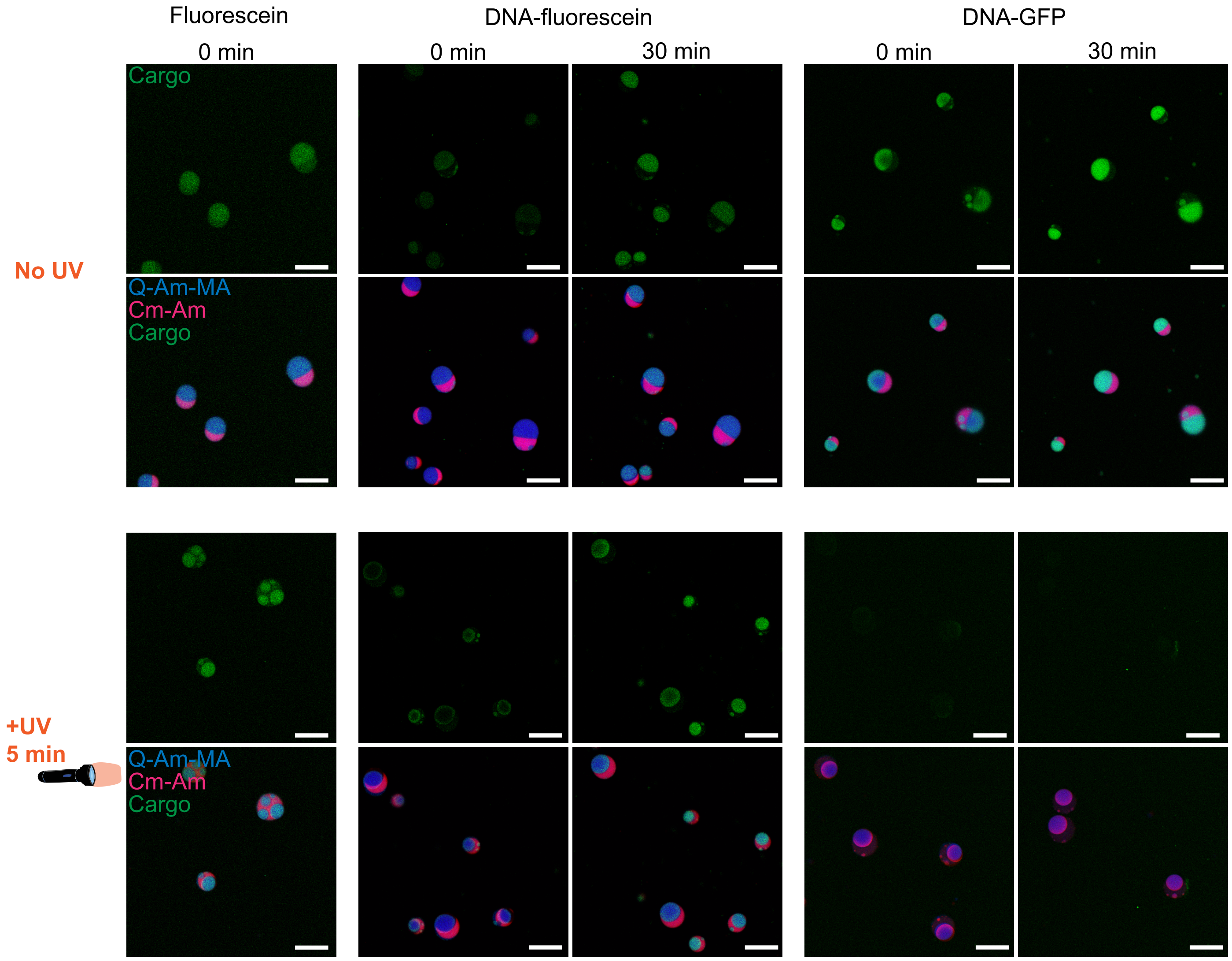

Figure S9. Cargo uptake in multiphase coacervates with and without Q-amylose-methacrylate crosslinking. 100 nM of three differently sized cargo molecules were added after coacervate formation, and uptake in the coacervates, based on favorable interactions like a complementary charge or DNA-interactions, was followed. The small molecule fluorescein is almost immediately taken up in both crosslinked and non-crosslinked coacervates. The intermediately sized DNA-fluorescein (38nt ssDNA, complementary to the ss-DNA used for the multiphase separation), is only taken up after ∼30 minutes in the non-crosslinked coacervates. In the crosslinked coacervates, uptake is also observed after 30 minutes, however, for the larger coacervates the DNA-fluorescein signal is not homogeneous yet: the signal is higher on the periphery of the inner dense phase. For the large DNA-GFP (∼27 kDa), uptake is only observed for the non-crosslinked coacervates. When crosslinked, the DNA-GFP is not able to localize in the coacervates. This shows that cargo uptake is limited for larger cargos in crosslinked coacervates, as can be expected after forming an Q-Am-MA network. Scale bars are 20μm.
